# Experience of urologists, oncologists and nurse practitioners with mainstream genetic testing in metastatic prostate cancer

**DOI:** 10.1038/s41391-024-00925-w

**Published:** 2024-12-05

**Authors:** Michiel Vlaming, Margreet G. E. M. Ausems, Lambertus A. L. M. Kiemeney, Gina Schijven, Harm H. E. van Melick, M. Arjen Noordzij, Diederik M. Somford, Henk G. van der Poel, Carl J. Wijburg, Bart P. Wijsman, Robert J. Hoekstra, Reindert J. A. van Moorselaar, Bart P. J. van Bezooijen, Richard P. Meijer, Martijn B. Busstra, H. Pieter van den Berg, Debbie G. J. Robbrecht, Benjamin H. J. Doornweerd, Eveline M. A. Bleiker, Inge M. van Oort

**Affiliations:** 1 Department of Genetics, Division Laboratories, Pharmacy and Biomedical Genetics, University Medical Center Utrecht, Utrecht, The Netherlands; 2 Department for Health Evidence, Radboud university medical center, Nijmegen, The Netherlands; 3 Department of Urology, Radboud university medical center, Nijmegen, The Netherlands; 4 Department of Urology, St. Antonius Hospital, Utrecht-Nieuwegein, The Netherlands; 5 Department of Urology, Spaarne Gasthuis, Hoofddorp, The Netherlands; 6 Department of Urology, Canisius Wilhelmina Ziekenhuis, Nijmegen, The Netherlands; 7 Department of Urology, The Netherlands Cancer Institute, Amsterdam, The Netherlands; 8 Department of Urology, Amsterdam UMC, Vrije Universiteit Amsterdam, Amsterdam, The Netherlands; 9 Department of Urology, Rijnstate Hospital, Arnhem, The Netherlands; 10 Department of Urology, Elisabeth-TweeSteden Hospital, Tilburg, The Netherlands; 11 Department of Urology, Catharina Hospital, Eindhoven, The Netherlands; 12 Department of Urology, Meander Medical Center, Amersfoort, The Netherlands; 13 Department of Oncological Urology, University Medical Center Utrecht, Utrecht, The Netherlands; 14 Department of Urology, Franciscus Gasthuis, Rotterdam, The Netherlands; 15 Department of Internal Medicine, Tergooi Medical Center, Hilversum, The Netherlands; 16 Department of Medical Oncology, Erasmus Medical Center, Rotterdam, The Netherlands; 17 Department of Urology, University Medical Center Groningen, Groningen, The Netherlands; 18 Division of Psychosocial Research and Epidemiology, The Netherlands Cancer Institute, Amsterdam, The Netherlands; 19 Department of Clinical Genetics, The Netherlands Cancer Institute, Amsterdam, The Netherlands; 20 Department of Clinical Genetics, Leiden University Medical Center, Leiden, The Netherlands

**Keywords:** Cancer genetics, Prostate cancer

## Abstract

**Background:**

International guidelines recommend germline genetic testing for men with metastatic prostate cancer. If offered to all patients by genetic healthcare professionals, there will be insufficient capacity to cope with the high patient numbers. In a mainstreaming pathway, non-genetic healthcare professionals (ngHCPs) discuss and order germline genetic testing instead of referring patients to genetic healthcare professionals. We aimed to evaluate the experience of ngHCPs with pre-test genetic counselling and to explore the feasibility from the ngHCPs’ perspective.

**Methods:**

We carried out a prospective cohort study in 15 hospitals in the Netherlands. All participating ngHCPs (i.e. urologists, medical oncologists, specialist nurses and nurse practitioners) completed an online training module of 45 min. The ngHCPs completed a questionnaire both before the training and at three and nine months after it. Paired analyses were used to compare the first with the last questionnaires on attitude, confidence in the ability to discuss and order germline genetic testing, and perceived and actual knowledge of genetics and genetic testing.

**Results:**

167 ngHCPs were invited to participate of whom 69 completed the first questionnaire and started or completed the last one. They had a positive attitude towards offering genetic testing themselves. After nine months of providing pre-test genetic counselling, significantly more ngHCPs considered mainstreaming helpful (94% after versus 81% before, *p* = 0.01). Both perceived and actual knowledge increased significantly. Pre-test genetic counselling took less than 10 minutes for 82% of ngHCPs and the majority (88%) were in favour of continuing the mainstream pathway. Only six participating ngHCPs considered mainstreaming possible without completing a training module beforehand.

**Conclusions:**

After completing a short online training module, ngHCPs feel well-prepared to discuss germline genetic testing with metastatic prostate cancer patients.

## Introduction

International guidelines recommend germline genetic testing for men with metastatic prostate cancer (mPCa) [1–3]. Pathogenic germline variants found in high-risk cancer predisposition genes may have important consequences for a patient and his relatives, and germline genetic testing should therefore be accessible to all eligible patients. Patients with metastatic castration-resistant prostate cancer and a pathogenic germline or somatic variant in one of the *BRCA* genes might benefit from treatment with PARP inhibitors [4–6]. Patients with a pathogenic germline variant in *BRCA2* also have an increased risk of developing breast cancer [7]. In addition, relatives of men with a pathogenic germline variant in one of the genes that are related to breast cancer in women (*BRCA1*, *BRCA2*, *ATM*, *CHEK2*, *PALB2*) might benefit from genetic testing of the index patient. They may carry the same variant and are therefore at increased risk of developing cancer, e.g. breast, ovarian, prostate and pancreatic cancer, depending on the gene involved [8–10].

In the traditional genetic testing pathway, patients are referred to a genetic healthcare professional who discusses and requests genetic testing. If germline genetic testing were to be carried out in all patients with mPCa, the traditional genetic counselling and testing pathway is not prepared for such large numbers. An alternative approach would be a mainstream genetic testing pathway [11] in which pre-test genetic counselling is provided by a non-genetic healthcare professional (ngHCP) instead of a genetic healthcare professional. Only patients with a pathogenic germline variant or a relevant family history (e.g. breast or ovarian cancer) undergo extensive post-test genetic counselling with a genetic healthcare professional.

The most extensive experience with mainstream genetic testing has been gained in ovarian and breast cancer care, almost always after concise training for the ngHCPs [12–20]. So far, there are only three studies that implemented mainstream genetic testing in patients with prostate cancer [21–23]. These studies assessed patients’ experience, but only one also assessed the experience in a small group of medical oncologists and fellows [21]. As urologists and nurses (specialists and practitioners) also have key roles in prostate cancer care, it is important that they also endorse this alternative pathway. This has not yet been evaluated.

We therefore implemented a mainstreaming pathway for mPCa patients and aimed 1) to assess the attitudes of urologists, medical oncologists and nurses (specialists and practitioners) towards this pathway; 2) to assess ngHCPs’ knowledge of genetics in prostate cancer and self-efficacy towards discussing and ordering genetic testing; and 3) to determine the feasibility of incorporating the mainstreaming pathway in daily practice from the ngHCPs’ perspective.

## Patients and methods

### Study procedure

In this prospective cohort study, ngHCPs discussed and ordered germline genetic testing for patients with mPCa. The study was conducted in 15 hospitals in the Netherlands and the study procedures were described in detail in our study protocol [24]. The University Medical Center Utrecht’s Institutional Review Board determined that the Dutch Medical Research Involving Human Subjects Act does not apply to this questionnaire study and official approval from an ethics committee is therefore not required.

To educate ngHCPs about the basics of genetic counselling and genetic testing, they were invited to complete an online training module of 45 min, which included among others a simulated conversation with a patient. An overview of topics in the training module are published priorly in the study protocol [24]. After completion, they had a meeting with the study researcher (MV) to go over the mainstreaming manual. Three questionnaires were sent to ngHCPs; a first questionnaire (T0) before accessing the online training, a second questionnaire to detect practical issues three months after completing the training (T1) and a third questionnaire six months later (T2). Healthcare professionals who did not complete the training within 3 months received two reminders and if they did not respond, an invitation was sent to fill in a short questionnaire (Questionnaire B), adapted from Bokkers et al., to assess their reasons for not attending or not completing the module [12].

### Participants

Healthcare professionals (urologists, medical oncologists, specialist nurses and nurse practitioners) were invited if they were involved in prostate cancer care at one of the 15 participating hospitals and did not have a background in clinical genetics.

### Outcomes

The study variables for each questionnaire and the origins of the questions or statements used are summarized in Supplementary Table S1. The questionnaires consisted of statements about attitudes towards and self-efficacy of mainstream genetic testing, partially based on previous questionnaires and partially self-developed [12, 16]. In addition, questions about perceived knowledge of genetics and genetic testing, questions about actual knowledge of genetics and genetic testing and questions about the feasibility of mainstream genetic testing (e.g. time investment of mainstreaming and evaluation of online training module) were also partially based on previous questionnaires and partially self-developed [12, 25]. The statements about attitudes, self-efficacy and perceived knowledge were rated using a 5-point Likert scale (strongly disagree to strongly agree). The results of Questionnaire T1 will not be discussed in detail in this paper because it was only used for detecting practical issues.

### Statistics

Descriptive statistics were used to describe the ngHCPs’ characteristics and evaluation of feasibility questions. In the statements where a 5-point Likert scale was used and for knowledge questions we performed paired analysis between the T0 and T2 questionnaires with the Wilcoxon signed-rank test. Only ngHCPs who completed both questionnaires (T0 and T2) were included in these analyses. In the 5-point Likert scale, ‘agree’ and ‘strongly agree’ were recoded as positive and the other answers (‘not agree nor disagree’, ‘disagree’ and ‘strongly disagree’) were recoded as negative. To determine whether the ngHCPs included in the paired analysis were a good representation of the entire group, characteristics of ngHCPs, and answers to the T0 questionnaire, were compared between the ngHCPs who did and who did not complete the T2 questionnaire.

## Results

**Fig. 1:**
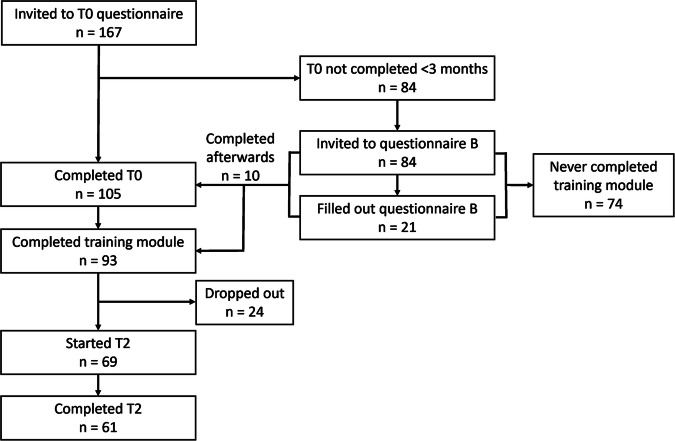
Flow diagram of participating non-genetic healthcare professionals.

Of the 167 ngHCPs invited, 93 (55%) completed the training module (Fig. 1). Of those, 61 completed the T2 questionnaire in full and 8 ngHCPs started T2 without completing it. Characteristics of participating ngHCPs who completed questionnaire T0 and started or completed T2 are summarized in Table 1. The vast majority (60/69, 87%) of the ngHCPs indicated that they had discussed or requested genetic testing with their patients. Characteristics of non-participants (*n* = 74) are shown in Supplementary Table S2 and were comparable to those of the participants, except for the significantly higher percentage of disciplines categorized as ‘other’. Twenty-one non-participants filled out Questionnaire B. The 24 ngHCPs who dropped out from the study did not differ significantly in baseline characteristics and answers to the T0 questionnaire from those who filled out the T2 questionnaire (data not shown).

**Table 1:** Characteristics of non-genetic healthcare professionals who completed T0 and started or completed the T2 questionnaire, *n* = 69.

Characteristics	Total, *n* (%)
Mean age (years), min–max	46 (29-61)
Sex	
Male	38 (55%)
Female	31 (45%)
Discipline	
Urologist	30 (44%)
Medical oncologist	14 (20%)
Nurse practitioner or physician assistant	15 (22%)
Nurse or specialist nurse	9 (13%)
Other	1 (1%)
Years in prostate cancer care	
<5	13 (19%)
5–10	17 (25%)
10–15	12 (17%)
>15	27 (39%)
Hospital type	
Academic	29 (42%)
Non-academic	40 (58%)

### Attitude, self-efficacy, perceived knowledge

Participating ngHCPs had a positive attitude towards offering germline genetic testing themselves, both before implementing the mainstreaming pathway and nine months afterwards, with 88% agreeing or strongly agreeing before and 86% afterwards (Table 2). Seven ngHCPs switched from being positive at T0 to negative at T2 and stated that they did not have sufficient time (*n* = 2), that it was not one of their tasks (*n* = 2) because genetic testing yielded too little (*n* = 1) or for unspecified reasons (*n* = 2); five switched from being negative to being positive. After implementing the mainstreaming pathway, a significantly larger proportion of ngHCPs deemed it helpful that they could offer genetic testing themselves; 81% agreed or strongly agreed before implementation and 94% afterwards (*p* = 0.01). Only half of the ngHCPs felt mainstreaming necessary (48% before and 57% after implementing mainstreaming) and the large majority (78% before and 83% afterwards) stated that mainstreaming improved healthcare (*p* = 0.44). At T2, only 6 out of 61 ngHCPs (10%) considered mainstreaming possible without completing an online training module beforehand; 50 (82%) ngHCPs considered it possible after following an online training module only.

**Table 2:** Attitude, perceived knowledge and self-efficacy of non-genetic healthcare professionals before (T0) and nine months after (T2) completing the online training module, *n* = 69.

	T0 agree or strongly agree, *n* (%)	T2 agree or strongly agree, *n* (%)	*p*-value
Attitude			
I am positive about offering a genetic test myself	61 (88%)	59 (86%)	NS (0.56)
I find it helpful that a urologist, oncologist or nurse (specialists and practitioners) can discuss and order genetic testing	56 (81%)	65 (94%)	0.01
I find it necessary that a urologist, oncologist or nurse (specialists and practitioners) can discuss and order genetic testing	33 (48%)	39 (57%)	NS (0.26)
I find it an improvement in healthcare that a urologist, oncologist or nurse (specialists and practitioners) can discuss and offer genetic testing	54 (78%)	57 (83%)	NS (0.44)
It is important that patients have a choice of whether or not to have a genetic test performed^a^	59 (97%)	60 (98%)	NS (0.56)
It is important that all patients with prostate cancer can have genetic testing if there is an indication for it according to the guidelines^a^	57 (93%)	57 (93%)	NS (1.00)
It is important to pay attention to the psychosocial consequences of genetic testing when discussing genetic testing^a^	58 (95%)	60 (100%)	NS (0.08)
Urologists, oncologists and nurses (specialists and practitioners) are capable of offering pre-test genetic counselling and ordering genetic testing themselves after completing an online training module^a^	N/A	50 (82%)	N/A
Urologists, oncologists and nurses (specialists and practitioners) are capable of offering pre-test genetic counselling and ordering genetic testing themselves without completing an online training module^a^	N/A	6 (10%)	N/A
Perceived knowledge			
I understand the advantages and disadvantages of a genetic test^a^	36 (59%)	51 (84%)	0.002
I understand the importance of genetic testing for patients with metastatic prostate cancer^a^	49 (80%)	60 (98%)	<0.001
I understand the importance of genetic testing for family members of patients with metastatic prostate cancer^a^	44 (72%)	55 (90%)	0.002
Self-efficacy			
I am confident that I can discuss a genetic test with a patient with prostate cancer^a^	50 (82%)	52 (85%)	NS (0.62)
I am confident that I can discuss the advantages and disadvantages of a genetic test^a^	41 (67%)	50 (82%)	0.01
I am confident that I can order a genetic test myself^a^	50 (82%)	48 (79%)	NS (0.59)
I am confident that I can recognize psychosocial problems related to genetic testing in patients^a^	41 (67%)	44 (72%)	NS (0.44)
I am confident that I can explain what genetic testing in tumour tissue entails and what the differences are with genetic testing in blood samples^a^	46 (75%)	49 (80%)	NS (0.41)

*N/A* not applicable, *NS* not significant.

^a^*n* = 61 non-genetic healthcare professionals.

Perceived knowledge significantly increased after nine months of mainstreaming, as well as confidence in discussing the advantages and disadvantages of genetic testing (*p* = 0.01). Confidence in discussing genetic testing with a prostate cancer patient was high both before (82%) and after (85%) implementing the pathway.

### Knowledge

Participating ngHCPs showed a significant increase in knowledge (*p* < 0.001), with a mean total number of correct answers in the T0 of 10 out of 14 (SD 2.9) and 11 in the T2 (SD 2.0), (Supplementary Table S3). Questions with the largest increase in correct answers had to do with criteria for referring prostate cancer patients to the genetics department in routine healthcare.

### Feasibility of mainstream genetic testing

Providing pre-test genetic counselling took less than 10 minutes for the majority (82%) of ngHCPs, which was ‘better or much better than expected’ for 66% of the ngHCPs (Table 3). When taking discussing and ordering genetic testing together, 84% determined it feasible. After nine months’ experience with mainstream genetic testing, 88% (*n* = 53/60) of ngHCPs were in favour of continuing with the mainstreaming pathway.

**Table 3:** Time investment by participating non-genetic healthcare professionals in the questionnaire.

	Total, *n* (%)
How much time did you spend discussing genetic testing?^a^	
0–10 min	50 (82%)
10–20 min	10 (16%)
>20 min	1 (2%)
Is the amount of time that you spent discussing genetic testing what you expected? It was:^a^	
Better or much better than expected	40 (66%)
Neither better nor worse than expected	18 (30%)
Worse or much worse than expected	3 (5%)
How much time did you spend ordering genetic testing?^b^	
0–10 min	39 (85%)
10–20 min	5 (11%)
>20 min	2 (4%)
Is the amount of time that you spent ordering genetic testing what you expected? It was:^c^	
Better or much better than expected	28 (58%)
Neither better nor worse than expected	13 (27%)
Worse or much worse than expected	7 (15%)
Is the total time investment for discussing and/or ordering genetic testing feasible?^d^	
Yes	52 (84%)
No	6 (10%)
I don’t know	4 (6%)

^a^*n* = 61 (8 non-genetic healthcare professionals (ngHCPs) answered ‘not applicable’/answer was missing).

^b^*n* = 46 (23 ngHCPs answered ‘not applicable’/answer was missing).

^c^*n* = 48 (21 ngHCPs answered ‘not applicable’/answer was missing).

^d^*n* = 62 (7 ngHCPs answered ‘not applicable’/answer was missing).

### Evaluation of supporting materials and reasons for not completing the online training module

Nearly all (*n* = 60/61, 98%) ngHCPs found it helpful to receive information about genetic testing before providing pre-test genetic counselling. They received information through an online training module of 45 min, which was deemed clear or very clear by a large majority (*n* = 90/93, 97%) and highly useful (*n* = 91/93, 98%), (Supplementary Table S4). Additionally, an FAQ form was given, which 34/61 ngHCPs (56%) stated was useful. 54/61 ngHCPs (89%) found it helpful to give written information to their patients after discussing genetic testing.

Of the 21 ngHCPs who did not complete the online training module and filled out Questionnaire B, 13 (62%) did not try to log in, mainly because of time constraints. Three ngHCPs experienced technical problems that prevented them from completing the online training module.

## Discussion

To the best of our knowledge, this is the largest study that has examined the experiences of ngHCPs (i.e. urologists, medical oncologists, specialist nurses and nurse practitioners) with mainstream genetic testing in mPCa. We have shown that the majority of these ngHCPs are positive about offering germline genetic testing themselves to patients with mPCa and support continuing the mainstreaming pathway, with an online training module seen as a prerequisite for implementing the mainstreaming pathway. Moreover, ngHCPs are well prepared, have high perceived knowledge and self-efficacy for discussing genetic testing and they consider mainstreaming feasible. This may improve access to germline genetic testing.

Our mainstreaming pathway and online training module are based on prior studies in breast and ovarian cancer [12, 13]. In general, surgical oncologists, gynaecological oncologists and specialist nurses—the main ngHCPs in the studies previously mentioned—have more experience with genetic testing than urologists, the latter being the largest contributors to our study. Patients with mPCa have been eligible for genetic testing in the USA since 2018 [26] and in Europe since 2021 [27], whereas it has been common for breast and ovarian cancer for decades [28]. Despite the limited experience with genetic testing in mPCa, the large majority of ngHCPs are positive about providing pre-test genetic counselling. This is in line with previous studies among ngHCPs in breast and ovarian cancer care [14, 16, 18].

Until now, only three studies have shown data about mainstream genetic testing in prostate cancer [21–23] and only one assessed the ngHCPs’ experiences [21]. In two studies, ngHCPs were also trained with a short training course: one training item about germline genetic testing, pre-test counselling and study-specific information consisted of a face-to-face meeting of one hour [21]. The other—about genetic counselling, the importance of genetic testing and the current guidelines—consisted of a presentation of 30–60 min by study team members [23]. Our online training of 45 min consisted of basic genetic knowledge, indications for genetic testing, risks and benefits for patients and relatives and pre-test counselling information; this training was accredited. Our training added a simulated conversation and could be watched again, which quarter of the participating ngHCPs did. It is described in more detail in our study protocol [24]. An online training module was described as a prerequisite for implementing a mainstreaming pathway by nearly all of our participating ngHCPs. A positive influence on the level of knowledge of the online training module was observed, as more questions were answered correctly over time. This could be caused solely by the online training module or also because ngHCPs were more aware of genetics when offering genetic testing themselves. Based on existing scientific literature at the start of the study, we chose to only include patients with mPCa due to the highest prevalence of pathogenic germline variants. However, patients with mPCa, but patients with non-metastatic prostate cancer and relevant family history are also eligible for genetic testing [2]. In our training module, we explained the eligibility criteria for genetic testing for these patients and advised ngHCPs to refer these patients to the genetics department. This could raise awareness about genetics in prostate cancer and non-metastatic prostate cancer patients might therefore benefit as well. However, in the future, these patients may also be included in a mainstreaming pathway.

An interview study on barriers and facilitators of genetic counselling and germline testing in prostate cancer showed that many urologists felt a barrier towards conducting genetic testing due to time constraints [29]. However, in our study, 84% considered the time for discussing and ordering genetic testing feasible. The majority of ngHCPs (82%) were able to discuss genetic testing in less than 10 min and for 66% this was faster than expected. In previous ovarian, prostate and breast cancer studies, discussing genetic testing by ngHCPs was deemed feasible as well, mostly within the timeframe of a standard consultation [11, 14, 16, 18, 21]. The time that ngHCPs need to discuss genetic testing is less than genetic healthcare professionals [30], but this is acceptable as long as patients are given the key information to make an informed choice about whether or not to undergo genetic testing. A previous study comparing breast cancer patients’ experiences with pre-test counselling by ngHCPs or genetic healthcare professionals showed no significant differences [31]. Key topics in pre-test counselling are an explanation of the genes being tested and corresponding cancer risks, as well as the possible implications of genetic testing for a patient and his relatives [11]. To reduce the time required, not all trained ngHCPs need to perform both counselling and requesting genetic testing. We showed that physicians and nurses can divide the tasks (i.e., physicians counsel and a nurse requests or vice versa). In addition to or instead of mainstream genetic testing, other pre-test counselling options are being published or assessed that could reduce the burden on the Genetics department. These include video-based, web-based of telephone-based pre-test counselling [32–34].

### Limitations

Of the 167 ngHCPs who received an invitation, 93 (56%) completed the first questionnaire and online training module, which may have resulted in response bias. A large proportion (*n* = 21, 28%) of ngHCPs who did not participate were from ‘other’ disciplines, e.g. residents. They are only at the participating departments for a short period and therefore did not opt for the training. Moreover, not every ngHCP at a participating department needs to conduct mainstream genetic testing, as long as there are a few dedicated ngHCPs per department. It is known from previous studies that 3–5 per centre is sufficient for maintaining the mainstreaming pathway [12, 19–21]. In our study, an average of 5 ngHCPs per hospital discussed genetic testing with their patients.

Of the 93 who completed the first questionnaire, 69 ngHCPs (75%) started the last questionnaire and 61 (66%) completed it in full. We compared the answers from the dropouts against the other answers and did not find any significant differences in baseline characteristics, attitude, actual or perceived knowledge, or self-efficacy. It might be possible that response bias affected the results of our study. However, the ngHCPs who dropped out mainly consisted of ngHCPs who included none or only one patient (17 out of 24). Therefore, the result represents the findings among ngHCPs who had the largest experience with mainstream genetic testing.

This article does not cover the experiences of mPCa patients who received pre-test counselling from ngHCPs. However, this will be addressed in a subsequent article.

## Conclusion

Non-genetic healthcare professionals were positive towards mainstream genetic testing in patients with metastatic prostate cancer and found that the mainstreaming pathway was feasible. They supported the pathway, but only after online training.

## Supplementary information

Supplementary Material

## Data Availability

The data sets generated during the present study are available from the corresponding author upon reasonable request.
