# Supplementary material for: Experience of urologists, oncologists and nurse practitioners with mainstream genetic testing in metastatic prostate cancer
Source: Prostate Cancer Prostatic Dis. 2024 Dec 5;28(3):789–94. doi: 10.1038/s41391-024-00925-w (PMC12399427; doi:10.1038/s41391-024-00925-w)
Supplement: Supplementary file 1 — Supplementary Material [file 41391_2024_925_MOESM1_ESM.docx]

**Supplementary material**

Table S1. Study measures for each questionnaire 2

Table S2. Characteristics of non-genetic healthcare professionals who did not complete the online training module (n=74) 3

Table S3. Knowledge of non-genetic healthcare professionals before completing the online training module and 9 months after (n=62) 4

Table S4. Evaluation of the online training module (n=93) 6

# Table S1. Study measures for each questionnaire

|  |  | **Questionnaires** | | |
| --- | --- | --- | --- | --- |
| **Domain** | **Origin of questions or statements** | **T0** | **T1** | **T2** |
| Baseline characteristics |  | X |  |  |
| Attitude towards mainstream genetic testing (7 – 9 statements) | Bokkers *et al.* [1], George *et al.* [2] and self-developed | X |  | X |
| Self-efficacy of offering genetic testing themselves (5 statements) | Bokkers *et al.* [1] | X |  | X |
| Perceived knowledge of genetic testing (3 statements) | Bokkers *et al.* [1] | X |  | X |
| Actual knowledge of genetics and genetic testing (14 questions and statements) | Claes *et al.* [3], Bokkers *et al.* [1] and self-developed | X |  | X |
| Feasibility of mainstream genetic testing |  |  |  |  |
| - Referring patients to the genetics department (3 questions) | Bokkers *et al.* [1] | X |  |  |
| - Rating of online training module (3 – 5 questions) | Bokkers *et al.* [1] |  | X | X |
| - Experience with supporting material (manual, checklist for family history, FAQ and patient information) (2 – 9 questions) | Bokkers *et al.* [1] and self-developed |  | X | X |
| - Time investment in discussing and requesting genetic testing (6 – 7 questions) | Bokkers *et al.* [1] |  | X | X |
| - Reasons for not discussing genetic testing (1 – 2 questions) | Bokkers *et al.* [1] |  | X | X |
| - Communication with the Genetics department (3 questions) | Bokkers *et al.* [1] |  | X |  |
| - Discussing genetic test results (2 questions) | Bokkers *et al.* [1] |  |  | X |
| - Continuing mainstream genetic testing (3 questions) | Self-developed |  |  | X |

[1] Bokkers K, Bleiker E, Aalfs C *et al.* Surgical oncologists and nurses in breast cancer care are ready to provide pre-test genetic counseling. Annals of surgical oncology. 2023;30:3248-58.

[2] George A, Riddell D, Seal S *et al.* Implementing rapid, robust, cost-effective, patient-centred, routine genetic testing in ovarian cancer patients. Scientific Reports. 2016;6:1-8.

[3] Claes E, Evers‐Kiebooms G, Boogaerts A, Decruyenaere M, Denayer L, Legius E. Communication with close and distant relatives in the context of genetic testing for hereditary breast and ovarian cancer in cancer patients. American Journal of Medical Genetics Part A. 2003;116:11-9.

# **Table S2. Characteristics of non-genetic healthcare professionals who did not complete the online training module** (n=74)

Other: Resident not in training, resident in training, physician-researcher, case manager, research coordinator, nuclear medicine physician and radiation therapist

| **Characteristics** | **Total, n (%)** |
| --- | --- |
| Sex  Male  Female | 40 (54%)  34 (46%) |
| Discipline  Urologist  Medical oncologist  Nurse practitioner or physician assistant  Nurse or specialist nurse  Other | 27 (36%)  13 (18%)  6 (8%)  7 (9%)  21 (28%) |
| Hospital type  Academic  Non-academic | 29 (39%)  45 (61%) |

# Table S3. Knowledge of non-genetic healthcare professionals before completing the online training module and 9 months after (n=62)

The correct answers are in bold

|  | **T0 correct, n (%)** | **T2 correct, n (%)** | **p-value** |
| --- | --- | --- | --- |
| If only genetic testing of tumour tissue has been done and a pathogenic variant is found in one of the breast cancer genes, then… (hereditary cancer has been proved / hereditary cancer has not been proved / **additional testing is needed to determine hereditary cancer** / I don’t know) | 51 (82%) | 48 (77%) | NS (0.44) |
| If only genetic testing of blood has been done and a pathogenic variant is found in one of the breast cancer genes, then… (**hereditary cancer has been proved** / hereditary cancer has not been proved / additional testing is needed to determine hereditary cancer / I don’t know) | 41 (66%) | 48 (77%) | NS (0.08) |
| If a pathogenic variant is found in one of the breast cancer genes, taking out disability or life insurance for family members will… (not be possible / be possible but always at a higher premium or with less favourable conditions / **be possible but sometimes at a higher premium or with less favourable conditions** / I don’t know) | 43 (69%) | 51 (84%) | 0.05 |
| In patients with metastatic prostate cancer, a pathogenic variant in one of the breast cancer genes (*BRCA1*, *BRCA2*, *CHEK2*, *PALB2*, *ATM*) is found in … (<5% / **5 – 15%** / 15 – 25% / >25% / I don’t know)* | 48 (79%) | 40 (66%) | NS (0.06) |
| Patients with localized prostate cancer are eligible for referral to the Genetics department with a first or second-degree relative with … (breast cancer diagnosed ≤40 / **≤50** / ≤60 / age does not matter / breast cancer is irrelevant / I don’t know)* | 31 (51%) | 39 (65%) | NS (0.10) |
| Patients with localized prostate cancer are eligible for referral to the Genetics department with a first or second-degree relative with … (ovarian cancer diagnosed at age ≤40 / ≤50 / ≤60 / **age does not matter** / ovarian cancer is irrelevant / I don’t know)* | 24 (39%) | 33 (54%) | NS (0.07) |
| Patients with localized prostate cancer are eligible for referral to the Genetics department with a first or second-degree relative with … (pancreatic cancer diagnosed at age ≤40 / ≤50 / ≤60 / **age does not matter** / pancreatic cancer is irrelevant / I don’t know)* | 22 (36%) | 37 (61%) | 0.003 |
| Patients with localized prostate cancer are eligible for referral to the Genetics department with a first or second-degree relative with … (prostate cancer diagnosed at age ≤40 / ≤50 / ≤60 / age does not matter / **prostate cancer is irrelevant** / I don’t know)* | 5 (8%) | 18 (30%) | 0.002 |
| All men who carry a pathogenic variant of a breast cancer gene will develop prostate cancer **(incorrect)*** | 53 (87%) | 60 (98%) | 0.02 |
| A man who does not carry a pathogenic variant of a breast cancer gene still can develop prostate cancer **(correct)*** | 57 (93%) | 60 (98%) | NS (0.18) |
| A man who carries a pathogenic variant of a breast cancer gene can pass this genetic mutation to his children **(correct)*** | 56 (92%) | 60 (98%) | NS (0.10) |
| Prostate cancer is always caused by hereditary factors **(incorrect)*** | 59 (97%) | 61 (100%) | NS (0.16) |
| A man can inherit a pathogenic variant of a breast cancer gene from his mother **(correct)*** | 55 (90%) | 58 (95%) | NS (0.32) |
| A man who has a sister with a mutation in a breast cancer gene has a 50% chance (1 in 2) of carrying the same variant **(correct)*** | 37 (61%) | 43 (71%) | NS (0.13) |

NS: not significant

* n=61 non-genetic healthcare professionals

# Table S4. Evaluation of the online training module (n=93)

|  | **Overall, n (%)** |
| --- | --- |
| Duration, n (%)  Too short or much too short  Exactly right  Too long or much too long | 1 (1)  61 (66)  31 (33) |
| Clarity, n (%)  Clear or very clear  Not clear, not unclear  Unclear or very unclear | 90 (97)  2 (2)  1 (1) |
| Usefulness, n (%)  Very or reasonably useful  Not useful or not useful at all | 91 (98)  2 (2) |
| Pace, n (%)  Too fast or much too fast  Exactly right  Too slow or much too slow | 0 (0)  66 (71)  27 (29) |
| Average rating out of 10 (range) | 7.9 (6 – 10) |
